# Similar risk of hospitalization and mortality for patients continuing and discontinuing LTOT

**DOI:** 10.1186/s12931-025-03417-0

**Published:** 2025-11-15

**Authors:** Filip Björklund, Andreas Palm, Josefin Sundh, Yet H. Khor, Magnus Ekström

**Affiliations:** 1 Department of Clinical Sciences Lund, Faculty of Medicine, Respiratory Medicine, Allergology, and Palliative Medicine, Lund University, Lund, Sweden; 2 Department of Medical Sciences, Allergy and Sleep Research, Uppsala University, Respiratory, Uppsala, Sweden; 3 Department of Respiratory Medicine, Faculty of Medicine and Health, Örebro University, Örebro, Sweden; 4 Respiratory Research@Alfred, School of Translational Medicine, Monash University, Melbourne, VIC Australia; 5 Department of Respiratory and Sleep Medicine, Austin Health, Heidelberg, VIC Australia; 6 Institute for Breathing and Sleep, Heidelberg, VIC Australia; 7 Faculty of Medicine, University of Melbourne, Melbourne, VIC Australia

**Keywords:** LTOT, Mortality

## Abstract

**Introduction:**

While the characteristics and medical management of patients treated with long-term oxygen therapy (LTOT) have transformed during the last decades, the evidence base for LTOT itself remains largely unchanged. This study aimed to compare the risk of hospitalization and death among hypoxemic patients discontinuing LTOT, with that of controls continuing therapy.

**Methods:**

This was a retrospective observational study of patients with LTOT included in the DISCOVERY cohort, using elements of target trial emulation. Patients who fulfilled LTOT criteria but discontinued treatment with LTOT within 90 days of initiation, not due to improving oxygenation or change of treatment modality, were identified. The risk of hospitalization and death as a composite outcome, and the risks of individual outcomes of hospitalization and death, were compared with age-, sex- and disease type-matched controls who continued LTOT, using Cox regression adjusted for confounders.

**Results:**

In total, 79 patients discontinuing LTOT and 395 controls were analyzed, both groups with a mean age of 73 (SD ± 11) years, 72% females, 65% underlying COPD. In adjusted Cox regression models, discontinuing LTOT was not associated with an increased risk of hospitalization or death (hazard ratio (HR) 1.0 95% Confidence interval (CI) 0.78–1.3), hospitalization (HR 0.99 95% CI 0.75–1.3), or death (HR 0.79 95% CI 0.61-1.0).

**Conclusion:**

In this study, patients fulfilling LTOT initiation criteria who discontinued therapy were not found to be at an increased risk of hospitalization or death, suggesting equipoise for a randomized trial of LTOT discontinuation or non-initiation in selected patient groups.

**Supplementary Information:**

The online version contains supplementary material available at 10.1186/s12931-025-03417-0.

## Introduction

Long-term oxygen therapy (LTOT) is an established treatment method for prolonging life in patients with chronic obstructive pulmonary disease (COPD) and severe hypoxemia [1, 2]. Use of LTOT is common [3], and contributes both to significant societal costs [4] and to a considerable burden, including adverse effects, for the individual [5, 6].

The evidence base for LTOT rests on two randomized controlled trials (RCTs) performed in the late 1970 s [1, 2]. The studies included COPD patients of predominantly male sex with a mean age of 65 years, both demonstrating a mortality reduction by up to 50% at three and two years after treatment initiation, respectively [1, 2].

While no RCT has since reexamined the effect of LTOT on mortality among patients with severe hypoxemia, rapid development has been seen in the medical management of chronic respiratory disease, and in the characteristics of the treated population [7, 8]. Recent trials have also questioned the effects of LTOT on clinical outcomes in contemporary patients, by showing both a lack of benefit among patients with moderate hypoxemia [9], and non-superiority of LTOT prescribed for 24 rather than 15 h per day in patients with severe hypoxemia [10].

In this paper, we aimed to evaluate the clinical utility of LTOT for severe hypoxemia in a modern patient population, by studying the risks of hospitalization and death among patients discontinuing treatment.

## Methods

This was a longitudinal analysis of the national DISCOVERY cohort [11, 12], emulating an open-label randomized target trial of LTOT discontinuation within 90 days of initiation versus continuation among patients with severe hypoxemia, estimating the treatment effect on mortality and hospitalization in an intention-to-treat population.

The DISCOVERY database is based on the Swedish National Registry for Respiratory Failure (Swedevox), which covers about 90% of all patients who have started LTOT in Sweden since 1987 [13, 14]. Patient baseline data from Swedevox were cross-linked with data on baseline comorbidities from the National Patient Registry, and data on hospitalizations and deaths from the Patient Registry and Cause of Death Registry respectively [15–18]. The study protocol was approved by the Swedish Ethical Review Authority, Dnr 2018/51, and results are reported in accordance with the TARGET statement [19].

First, eligible patients in the DISCOVERY cohort were identified as: (1) aged ≥ 18 years; (2) initiating LTOT with a registered PaO_2_ on ambient air < 8 kPa (59 mmHg) between 1987 and 2023; and (3) surviving up to a landmark date 90 days after LTOT initiation. Second, eligible patients who discontinued LTOT within 90 days of starting the treatment were identified. The cause of discontinuation were categorized in the registry as “no longer wanted/unknown” from a list also including the options “no longer needed”, “switch to other treatment form”, and “death”, thus indicating continued severe hypoxemia. Third, each patient who discontinued LTOT was matched to five eligible control patients who did not discontinue LTOT during the study period on: (1) treatment cause (COPD or non-COPD); (2) sex; and (3) age (± 2 years).

The primary composite outcome of all-cause hospitalization and death, as well as separate risks of hospitalization and death, were assessed from LTOT initiation to the first respective event, or end of study (October 2023) if no event occurred. Associations between LTOT discontinuation and outcomes were analyzed using Kaplan-Meier curves and Cox regression models. All regression models were analyzed crude and adjusted for other potential predictors of mortality (LTOT start year, body mass index, underlying disease, PaO_2_ and PaCO_2_ on room air and oxygen, prescribed oxygen dose, smoking history, Charlson comorbidity index, and performance status) [20–23]. Proportional hazards assumptions were confirmed using log-log plots. Missing covariates in adjusted analyses were imputed using multiple imputation by chain equation. The risk of hospitalization was also analyzed using Fine-Gray regression with death as a competing event, which yielded similar findings.

## Results

We included 79 patients discontinuing LTOT and 395 matched patients who continued LTOT in analyses (suppelemental figure S1), with a median follow-up of 482 days (IQR 163-1,118). Baseline characteristics were similar between groups, and between matched patients and patients in DISCOVERY who fulfilled inclusion criteria but were not matched, with the exception of a somewhat smaller proportion of females among non-matched patients, and a higher proportion of portable equipment use among matched patients continuing LTOT (Table 1, supplemental table S1, supplemental figure S2). Patients discontinuing LTOT did so after a median time on LTOT of 35 days (IQR 14–66).

**Table 1:** Baseline characteristics of patients voluntarily discontinuing LTOT and matched controls

	Patients discontinuing LTOT *n* = 79	Matched patients continuing LTOT *n* = 395	All patients fulfilling inclusion criteria in the DISCOVERY cohort* n = 20,032
Age, years (mean, SD)	73.3 (10.7)	73.1 (10.5)	73.6 (9.0)
Female (*n*, %)	57 (72%)	285 (72%)	11,196 (56%)
BMI, kg/m2 (mean, SD)	24.0 (6.0)	24.6 (6.6)	24.9 (6.5)
Underlying disease type (*n*, %)			
COPD	51 (65%)	255 (65%)	13,114 (65%)
Non-COPD	28 (35%)	140 (35%)	6,918 (35%)
ILD	11 (14%)	51 (13%)	3,158 (16%)
Pulmonary vascular disease	5 (6%)	26 (7%)	943 (5%)
Other	12 (15%)	63 (16%)	2817 (14%)
PaO _2_ on ambient air (mean, SD)	6.4 (0.83) kPa/ 48 (6.2) mmHg	6.5 (0.86) kPa/ 49 (6.5) mmHg	6.5 (0.81) kPa/ 49 (6.1) mmHg
PaO _2_ on oxygen (mean, SD)	8.7 (0.96) kPa/ 65 (7.2) mmHg	8.8 (1.2) kPa/ 66 (9.0) mmHg	8.8 (1.3) kPa/ 66 (9.8) mmHg
PaCO _2_ on ambient air (mean, SD)	6.3 (1.2) kPa/ 47 (9.0) mmHg	6.0 (1.3) kPa/ 45 (9.8) mmHg	6.0 (1.3) kPa/ 45 (9.8) mmHg
PaCO _2_ on oxygen (mean, SD)	6.4 (1.3) kPa/ 48 (9.8) mmHg	6.4 (1.4) kPa/ 48 (10.5) mmHg	6.3 (1.3) kPa/ 47 (9.8) mmHg
PaO2 ≤ 7.4 kPa on room air (*n*, %)	72 (91%)	338 (86%)	17,525 (87%)
PaO2 7.4–7.9.4.9 kPa on room air without documented polycythemia or edema	4 (5%)	34 (9%)	1,309 (7%)
Prescribed oxygen dose, L/min (mean, SD)	1.5 (0.72)	1.7 (0.98)	1.7 (1.1)
Prescribed oxygen 24 h/d (*n*, %)	15 (19%)	92 (23%)	4,701 (23%)
FEV1, L (mean, SD)	1.0 (0.96)	0.94 (0.66)	1.0 (0.62)
FEV1% predicted (mean, SD) **	43 (30)	45 (27)	44 (24)
Hemoglobin (g/dL)	14 (1.5)	14 (1.8)	14 (1.7)
Presence of peripheral edema	15 (19%)	103 (26%)	5,105 (25%)
Portable oxygen equipment prescribed	17 (22%)	177 (45%)	9,956 (50%)
Liquid oxygen prescribed	2 (3%)	9 (2%)	496 (2%)
Smoking status (*n*, %)			
Ever-smoker	64 (81%)	303 (77%)	16,081 (80%)
Never-Smoker	10 (13%)	64 (16%)	2,708 (14%)
Charlson Comorbidity Index (*n*, %)			
0	7 (9%)	26 (7%)	1,437 (7%)
1–2	44 (56%)	241 (61%)	11,727 (59%)
>2	28 (35%)	128 (32%)	6,868 (34%)
WHO Performance status (*n*, %)			
0	3 (4%)	18 (5%)	1,166 (6%)
1	27 (34%)	161 (41%)	8,686 (43%)
2	20 (25%)	130 (33%)	5,864 (29%)
3	17 (22%)	44 (11%)	2,073 (10%)
4	2 (3%)	4 (1%)	185 (1%)

*1) age ≥ 18 years; 2) initiation of LTOT with a registered PaO2 < 8 kPa between 1987 and 2023; and 3) survival up to a landmark date 90 days after treatment initiation. Only patients discontinuing LTOT and matched controls are included in analyses

** According to Berglund [25]

*Abbreviations*: *BMI* Body Mass Index, *COPD* chronic obstructive pulmonary disease, *dL* deciliter, *FEV1* Forced expiratory volume in one second, *g* Gram, *IQR* Interquartile range, *kPa* Kilopascal, *LTOT* Long-term oxygen therapy, *PaCO2* Partial pressure of carbon dioxide, *PaO2* Partial pressure of oxygen, *SD* Standard deviation, *WHO* World health organization

There was no significant difference in the median time to first hospitalization between patients discontinuing LTOT (0.27 years; [95% CI 0.16–0.57]) and controls (0.41 [0.32–0.56]), nor in the median survival time (2.5 [1.5–3.4] vs. 2.3 [2.0–2.6.0.6] years).

**Fig. 1:**
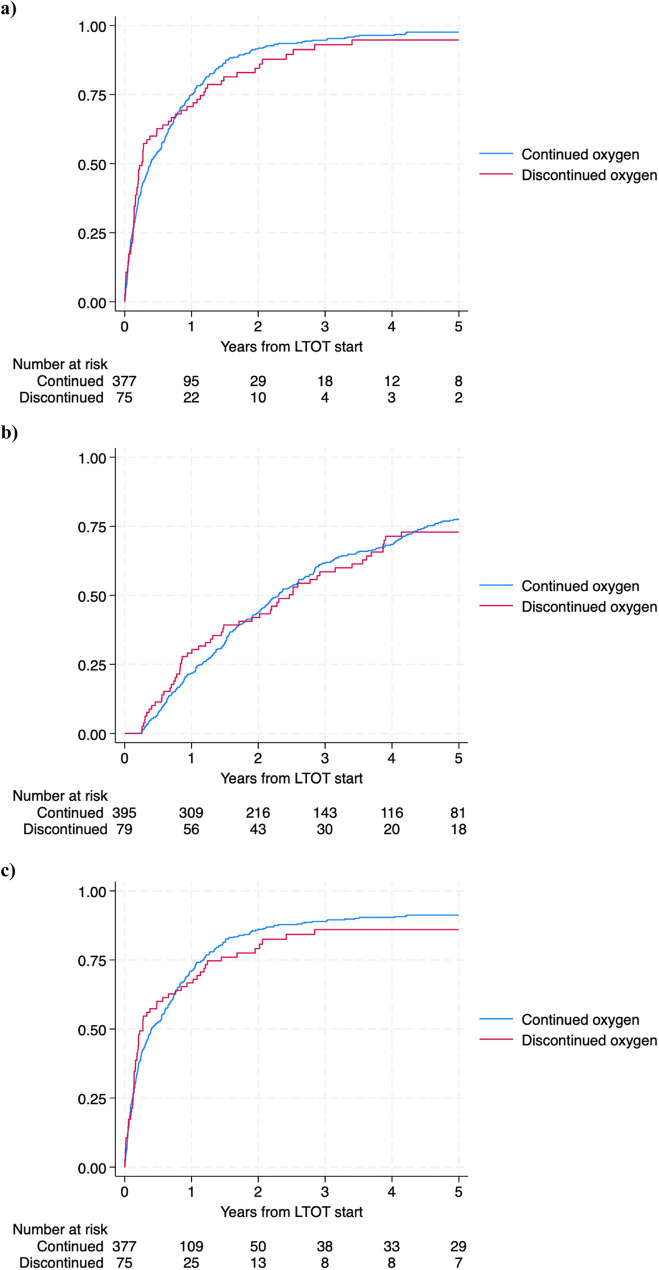
Kaplan-Meier curves representing probability of (**a**) hospitalization and death; (**b**) death; (**c**) hospitalization

Discontinuing LTOT was not associated with an increased risk of the composite outcome of hospitalization and death (adjusted HR 1.0 [0.78–1.3] for discontinuation), hospitalization (adjusted HR 0.99 [0.75–1.3]), or death (adjusted HR 0.79 [0.61–1.0.61.0]) in neither crude nor adjusted regression models (Fig. 1a and c; Table 2).

**Table 2:** Hazard ratios for composite primary event (hospitalization or death), hospitalization, and death

Outcome	Discontinuing vs. continuing LTOT, Hazard ratio (95% CI)	
	Crude model	Adjusted model
Composite outcome	0.95 (0.74–1.2)	1.0 (0.78–1.3)
Hospitalization (Cox model)	0.93 (0.71–1.2)	0.99 (0.75–1.3)
Hospitalization (Fine-Gray model)	0.93 (0.69–1.2)	0.99 (0.73–1.3)
Overall mortality	0.87 (0.67–1.1)	0.79 (0.61–1.0.61.0)

Estimates by Cox regression where not specified. Models analyzed crude and adjusted for LTOT start year, BMI, partial pressure of oxygen and carbon dioxide on room air and oxygen, prescribed oxygen dose, smoking history, CCI (as a continuous variable), and WHO performance status. Missing covariates in adjusted models were imputed using multiple imputation by chain equation. Control patients were matched on age, gender and underlying disease type. Lower HR favors discontinuation of LTOT

*Abbreviations*: *BMI* body mass index, *CCI* Charlson comorbidity index, *CI* Confidence interval, *LTOT* Long-term oxygen therapy, *WHO* World Health Organization

## Discussion

The main finding of this study is that no increased risk of hospitalization or death was observed for hypoxemic patients who discontinued LTOT, compared to controls continuing treatment. While the mechanism by which LTOT has been shown to extend life in some patients remains unknown, this effect may be partly time-dependent, either through the preservation of pulmonary hemodynamics [2, 10, 24], or through prevention of ischemic end-organ injury. As improved medical management available today likely defers the advent of hypoxemia and its sequelae such as overt heart failure to a later stage in respiratory disease [14], such therapeutic effects of LTOT would then not become apparent before death occurs related to advanced age, multimorbidity and frailty [8].

Strengths of this study include primarily the high degree of similarity between patients discontinuing and continuing LTOT (with the notable exception of use of portable oxygen equipment), allowing RCT emulation, while limitations include potential bias (where patients discontinuing LTOT may have done so due to compliance issues or adverse events related to the therapy, or may have had their cause for treatment discontinuation misclassified by the clinical staff), the small sample size, and lack of data on adherence to therapy during use. As patients were included in the cohort during nearly four decades, intra-group differences in medical management and demographics may also affect the results, as might the sizeable portion of non-COPD patients not included in the original trials of LTOT.

We see a need for reexamining the utility of LTOT, where further RCTs may investigate non-initiation or discontinuation of LTOT among selected patients, where this study may provide an indication of clinical equipoise.

## Supplementary Information

Supplementary Material 1

## Data Availability

As stated by the Swedish Ethical Review Agency which approved the data collection and analysis presented in this article, data is not allowed to be shared publicly. When entering the study, participants were promised that personal data would not be shared publicly. According to Swedish law (2003:460) concerning research including humans, ethical permission in required to process data including humans. To access study data, ethical approval must be granted from the Swedish Ethical Review Authority (https://etikprovningsmyndigheten.se). Requests for data should then be made to the corresponding author of this paper or to Medicinska fakulteten, Lunds universitet, Box 117, 221 00 LUND, Swedish Phone: +46-46-222 00 00, email: info@med.lu.se.
