# Supplementary material for: Similar risk of hospitalization and mortality for patients continuing and discontinuing LTOT
Source: Respir Res. 2025 Nov 15;26:321. doi: 10.1186/s12931-025-03417-0 (PMC12619151; doi:10.1186/s12931-025-03417-0)

**SUPPLEMENTAL MATERIAL**

**Supplemental table S1** Number and proportion of missing values for baseline patient characteristics.

|  | **Patients discontinuing LTOT**  n=79 | **Matched patients continuing LTOT**  n=395 |
| --- | --- | --- |
| **Age** |  |  |
| Missing | 0 (0%) | 0 (0%) |
| **Female** |  |  |
| Missing | 0 (0%) | 0 (0%) |
| **BMI** |  |  |
| Missing | 36 (46%) | 123 (31%) |
| **Underlying disease type** |  |  |
| Missing | 0 (0%) | 0 (0%) |
| **PaO2 on ambient air** |  |  |
| Missing | 0 (0%) | 0 (0%) |
| **PaO2 on oxygen** |  |  |
| Missing | 7 (9%) | 24 (6%) |
| **PaCO2 on ambient air** |  |  |
| Missing | 1 (1%) | 3 (1%) |
| **PaCO2 on oxygen** |  |  |
| Missing | 7 (9%) | 22 (6%) |
| **PaO2 ≤7.4kPa on room air** |  |  |
| Missing | 0 (0%) | 0 (0%) |
| **Prescribed oxygen dose** |  |  |
| Missing | 0 (0%) | 1 (0.2%) |
| **Prescribed oxygen 24h/d** |  |  |
| Missing | 0 (0%) | 5 (1%) |
| **FEV1, L** |  |  |
| Missing | 39 (49%) | 123 (31%) |
| **FEV1% predicted** |  |  |
| Missing | 48 (61%) | 182 (46%) |
| **Hemoglobin** |  |  |
| Missing | 58 (73%) | 254 (64%) |
| **Presence of peripheral edema** |  |  |
| Missing | 35 (44%) | 168 (43%) |
| **Portable oxygen equipment prescribed** |  |  |
| Missing | 17 (22%) | 34 (9%) |
| **Liquid oxygen prescribed** |  |  |
| Missing | 0 (0%) | 0 (0%) |
| **Smoking Status** |  |  |
| Missing | 5 (6%) | 28 (7%) |
| **Charlson Comorbidity Index** |  |  |
| Missing | 0 (0%) | 0 (0%) |
| **WHO Performance status** |  |  |
| Missing | 10 (13%) | 41 (10%) |

**Abbreviations:** BMI = Body Mass Index, COPD = chronic obstructive pulmonary disease, FEV1 = Forced expiratory volume in one second, kPa = Kilopascal, LTOT = Long-term oxygen therapy, PaCO2 = Partial pressure of carbon dioxide, PaO2 = Partial pressure of oxygen, WHO = World health organization

**Supplemental figure S1** Study inclusion flowchart.


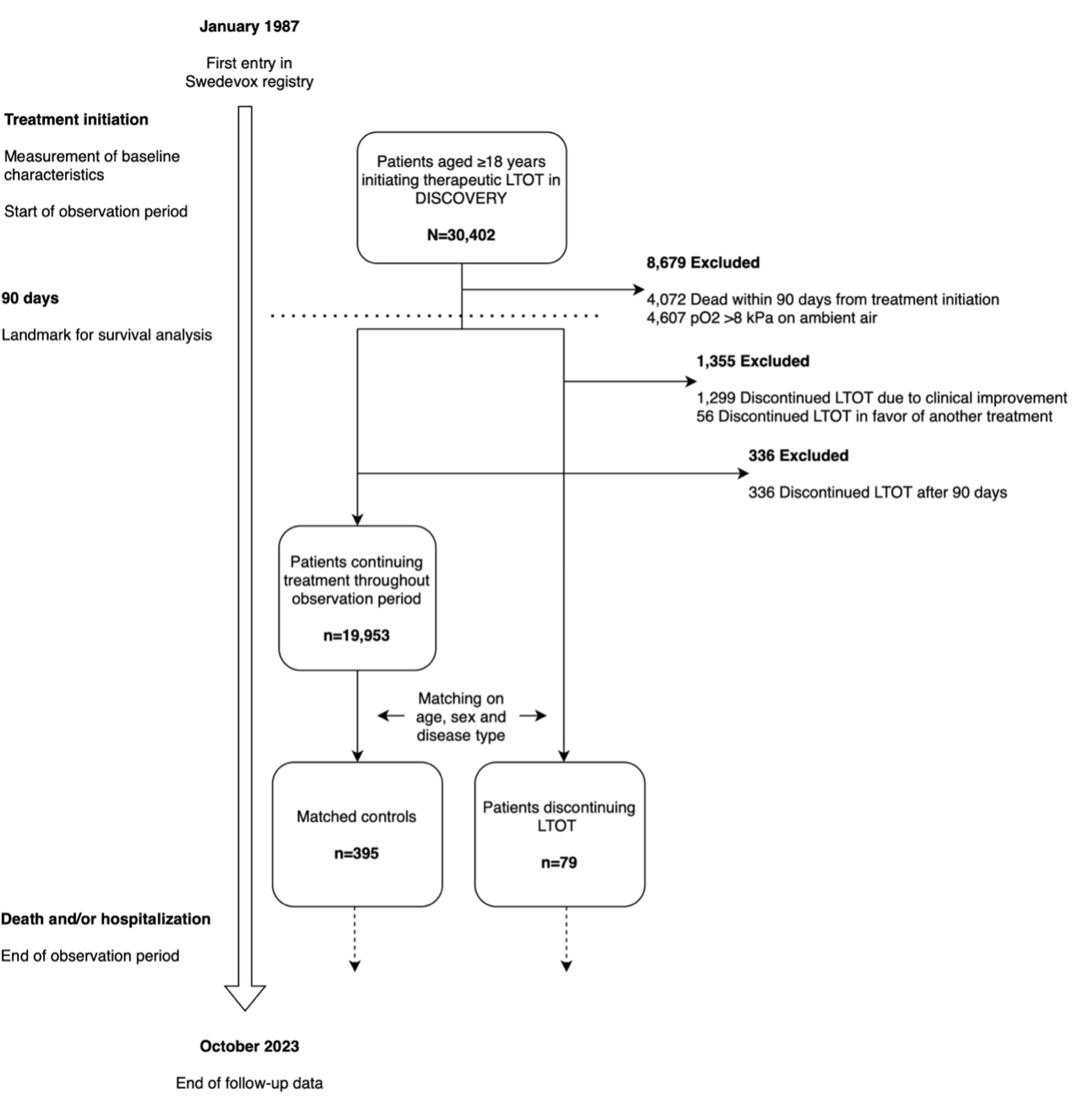


**Supplemental figure S2** Distribution of LTOT start year by study arm.


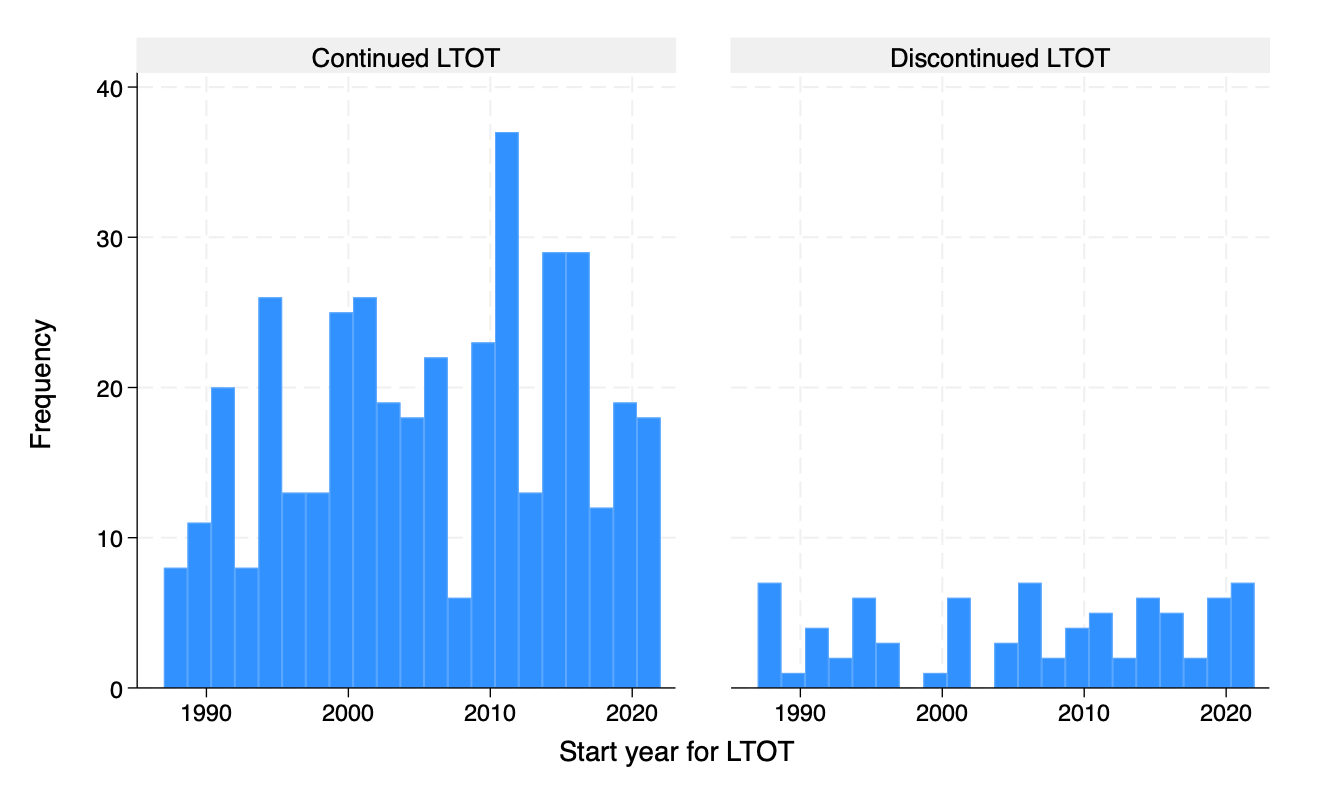

Supplement: Supplementary file 1 — Supplementary Material 1 [file 12931_2025_3417_MOESM1_ESM.docx]
